# Poor dental health and risk of pancreatic cancer: a nationwide registry-based cohort study in Sweden, 2009–2016

**DOI:** 10.1038/s41416-022-02018-8

**Published:** 2022-10-22

**Authors:** Jingru Yu, Alexander Ploner, Margaret Sällberg Chen, Ji Zhang, Gunilla Sandborgh-Englund, Weimin Ye

**Affiliations:** 1 Department of Medical Epidemiology and Biostatistics, Karolinska Institutet, 171 77 Stockholm, Sweden; 2 Department of Dental Medicine, Karolinska Institutet, 171 77 Stockholm, Sweden; 3 Tenth People’s Hospital, Tongji University, 200 072 Shanghai, China; 4 Academic Center for Geriatric Dentistry, Karolinska Institutet, 171 77 Stockholm, Sweden; 5 Department of Epidemiology and Health Statistics & Key Laboratory of Ministry of Education for Gastrointestinal Cancer, Fujian Medical University, 350 122 Fuzhou, China

**Keywords:** Risk factors, Inflammation

## Abstract

**Background:**

Previous studies have reported inconsistent results regarding the association between poor dental health and pancreatic cancer risk. This study aimed to assess this association using a well-functioning nationwide dental health registry in Sweden.

**Methods:**

Information of exposures (dental caries, root canal infection, mild inflammation, and periodontitis; the number of teeth) was ascertained from the Swedish Dental Health Register, and occurrence of pancreatic cancer was identified from both cancer and cause of death registries. Hazard ratios (HRs) were estimated using Cox models.

**Results:**

During a median of 7.2 years of follow-up, 10,081 pancreatic cancers were identified among 5,889,441 individuals. Compared with the healthy status, a higher risk of pancreatic cancer was observed in individuals with root canal infection, mild inflammation, and periodontitis in the <50 age group (*P* for trend <0.001). In the 50–70 age group, only the subgroup with periodontitis had an excess risk (multivariable-adjusted HR = 1.20, 95% confidence interval [CI] 1.11–1.29). No positive association with statistical significance was observed in the 70+ age group. Individuals with fewer teeth tended to have a higher risk in all age groups.

**Conclusions:**

Our results confirmed the association between poor dental health and pancreatic cancer risk, which warrants further studies on underlying mechanisms.

## Introduction

Pancreatic cancer is a lethal malignancy, with 5-year survival rate <9% [1]. Though the incidence is low, the number of new cases and of deaths from pancreatic cancer are predicted to increase regionally and globally due to ageing populations [2]. Established risk factors include old age, male sex, chronic pancreatitis, diabetes, obesity, tobacco smoking, and family history of pancreatic cancer [3, 4]. However, these risk factors explain only a fraction of pancreatic cancer cases, and other risk factors are largely unknown yet.

The oral cavity, as a gateway, including >700 species of bacteria, connects the gastrointestinal tract and the external environment [5]. Oral hygiene and lifestyle factors have significant impact on the host microbiome [6]. Oral microbiome dysbiosis can augment progression of dental diseases and consequent tooth loss. Dental caries (dental plaque) is initiated by the bacterial biofilm covering on the dental surface, without concurrent inflammation in surrounding tissues [7]. If left untreated, it can progress to pulpitis and apical periodontitis that result in local inflammation [8]. Periodontal diseases (gum diseases), such as gingivitis, mild or advanced periodontitis, and periimplantitis, induce an inflammatory response [9]. Gingivitis, the mildest form of periodontal disease, is caused by the bacterial biofilm that accumulates on teeth adjacent to the gums; it does not affect the underlying supporting structures of the teeth and is reversible. Unlike gingivitis, periodontitis is the advanced stage of gum disease and involves loss of alveolar bone around the teeth, ultimately leading to tooth loss if untreated [9]. Notably, emerging evidence indicates that myocardial infarction [10], diabetes [11], cancer risks [12–14], and cancer mortality [15] are related to chronic microbial imbalance and systemic inflammation.

To date, the evidence for the link between poor dental health and pancreatic cancer risk is not always consistent, partly due to variations in study population, exposure measurement, and/or selection on adjusted confounding factors, and a lack of statistical power (Supplementary Table 1). Consequently, we aimed to quantify the association between poor dental health, in particular inflammation related oral disorders, and the risk of pancreatic cancer based on the nationwide Dental Health Register in Sweden. This registry provides continuous and good quality monitoring of dental health conditions of the vast majority of Swedish adult population [16].

## Methods

### Study design

**Fig. 1:**
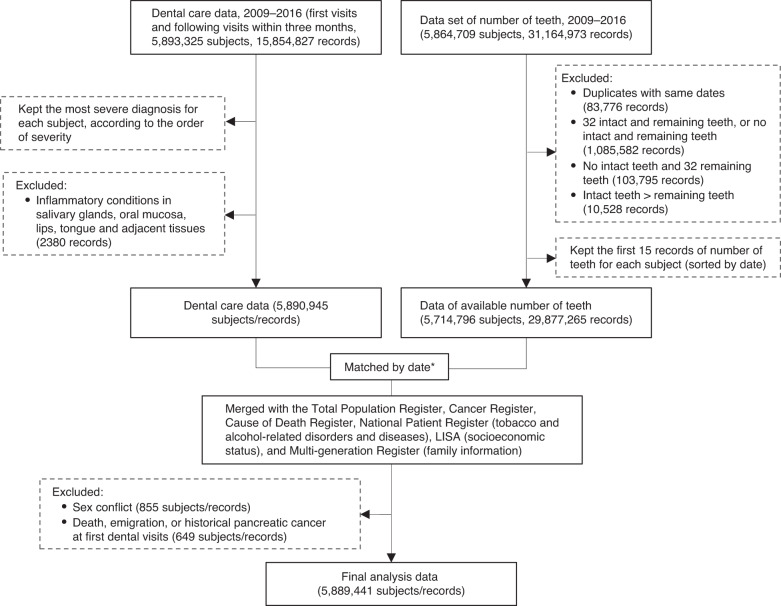
Flow chart for recruitment of the study population. *Records of the number of teeth were matched with dental care records by date of dental visit. If they were not matched by the same date, the number of teeth with the date that is closest to dental care date up to 6 months before or 5 years after was used.

We included a total of 5.9 million individuals older than 19 years with at least one dentist visit between 2009 and 2016. The unique personal identity numbers included in all Swedish registries were used to follow all individuals in this dental health cohort through linkages to the Swedish Cancer Register, Cause of Death Register, and Total Population Register. Each individual was followed from entry at the first dental visit until outcome occurrence, death, migration out of Sweden, or end of follow-up (December 31, 2016), whichever came first. After excluding subjects with pancreatic cancer, death and emigration at or before baseline, and conflicting information of sex between Total Population Register and Dental Health Register, we included 5,889,441 individuals with both information of dental diagnoses and the number of teeth in the final analysis cohort. The workflow for cohort assembly is shown in Fig. 1.

### Assessment of exposures

Based on dental care data, we classified oral health conditions in teeth and their supporting tissues caused by dental biofilm (layers of microorganisms attached to the supragingival or subgingival surface of a tooth) according to the severity of oral health damage and inflammation (Supplementary Table 2) [17] as healthy, caries, root canal infection, mild inflammation (including gingivitis, pericoronitis, stomatitis, mucositis, and other unspecific inflammatory conditions), and periodontitis (including mild to advanced periodontitis, and periimplantitis [18, 19]). Information on the number of teeth was also collected, and subjects were grouped according to the number of teeth at the first visit into categories (28–32, 25–27, 21–24, 15–20, and 1–14 teeth); patients in whom this information was missing were categorised as “unknown” (Supplementary Methods).

### Assessment of outcome

Reporting any diagnosis of cancer has been mandatory for clinicians, cytologists, and/or pathologists in Sweden since 1958, and the overall completeness of the Swedish Cancer Register has been estimated at >98% [20]. However, cancers without histopathological confirmation are less likely to be reported but may be reported to the Swedish Cause of Death Register, based on obligatory death certificates. Therefore, we ascertained pancreatic cancer cases not only from the Cancer Register through the International Classification of Diseases version 7 (ICD-7: 157), but also from the Cause of Death Register (underlying cause, ICD-7/8/9:157, ICD-10: C25), to compensate for underreporting of pancreatic cancer in the Cancer Register [21].

### Other covariates

Individuals’ socioeconomic background may serve as a potential confounder for the studied association, thus we obtained information about education level and family income from the Longitudinal Integration Database for Health Insurance and Labour Market Studies (LISA). Education level was classified as (a) low, if the highest schooling was primary education 9 years and below, (b) medium, if 2 or 3 years of secondary schooling, (c) high, if postsecondary education and above, and (d) unknown information. Family income at baseline was categorised into three levels: low, medium, and high among subjects aged 20–65 years and older than 65 years (retired) separately, using age-specific tertiles from the Swedish general population (Supplementary Table 3). Diagnoses of tobacco abuse, and chronic obstructive pulmonary disease (as a proxy for smoking, Supplementary Table 4), and alcohol-related disorders and diseases (as a proxy for alcohol drinking, Supplementary Table 4) were obtained from the National Patient Register by using ICD codes. With regard to family history of pancreatic cancer, all first-degree family members of individuals in this cohort were linked to the Swedish Multi-generation Register by personal identity numbers, and then linked to the Swedish Cancer Register and Cause of Death Register to capture all pancreatic cancer events. A Directed Acyclic Graph (DAG) for the list of potential confounders is shown in Supplementary Figure.

### Statistical analysis

We reported the baseline characteristics and dental health-related factors (dental health status and number of teeth categories) of all individuals by age at entry into cohort (>19 and <50, ≥50 and <70, and ≥70 years).

Cox proportional hazards models were fitted to calculate hazard ratios (HRs) with 95% confidence intervals (CIs), to examine the association between poor dental health and the risk of pancreatic cancer. We stratified age at baseline into the same three groups and fitted Cox models with attained age as the underlying time scale in each stratum. We selected healthy dental status and ≥28 teeth as the reference group, with adjustment for sex, calendar year of first visit, education, family income, family history of pancreatic cancer, smoking-related diseases, and alcohol-related disorders and diseases. Furthermore, each model included both dental health status and number of teeth, so that our main exposures were always adjusted for each other.

The proportional hazards assumption was assessed by a Schoenfeld residual-based test, and no violation was observed. The Cochran–Armitage trend test was used to evaluate a possible trend of the risk of pancreatic cancer across decreasing levels of dental health status (inflammation), and the number of teeth.

We estimated interactions between age at baseline and poor dental health, and between age at baseline and the number of teeth fit to the whole data (Supplementary Methods). These preliminary models suggested strong effect modification for both main exposures by age at baseline, and this was no longer the case after stratifying the data by age at baseline. Therefore, we continued with the separate Cox models across this study.

In a separate analysis, we included an interaction term between dental inflammation (inflammation group includes mild inflammation and periodontitis; no inflammation group includes healthy, caries, and root canal infection) and number of teeth in all three age-specific Cox models. This interaction was estimated by including a cross-product term between the binary dental inflammatory condition (having inflammation or not) and the indicator variables for categories of number of teeth, and the null hypothesis of no interaction was assessed using a likelihood ratio test.

In sensitivity analyses, we explored the effects of multiple measurements during follow-up (time-varying exposures): for each participant, the most severe diagnosis of dental health status in each calendar year was identified after baseline, and treated as a time-varying exposure. The follow-up period was split at progressive dates. Similarly, we identified any reduction in the number of teeth during follow-up, and also included it as a time-varying exposure with split-follow-up time in the three age-stratified Cox models. We also included a one-year lag in the Cox models as a sensitivity analysis, i.e. starting follow-up one year after the first visit, to minimise selection bias. Additionally, models were re-fitted while limiting the outcome to incident cases from the Cancer Register only, i.e. excluding cases from the Cause of Death Register, to assess the possible effect of selective reporting of pancreatic cancer.

Statistical analyses were performed using Stata (Version 14; Stata Corp, College Station, TX, USA), and data visualisation was performed using R (Version 3.6.1; R Foundation for Statistical Computing, Vienna, Austria). Two-sided *P* < 0.05 was considered statistically significant.

## Results

In the main analyses, 5,889,441 individuals with median follow-up of 7.2 years were included. The mean age at baseline was 53.7 years (Table 1). About 30% of all individuals had dental inflammatory conditions (mild dental inflammation or periodontitis), among whom more than one-third had periodontitis. Individuals with periodontitis at baseline were older, more likely to be smokers, and had fewer teeth. Similarly, individuals with fewer teeth (1–14) at baseline were older, had lower education level, family income, and more likely to be smokers and alcohol drinkers (Supplementary Tables 5 and 6).

**Table 1:** Distribution of characteristics and dental health-related factors by age at baseline in the cohort identified from the Swedish Dental Health Register, 2009–2016.

Characteristics	Age at baseline			
	>19 and <50 years	≥50 and <70 years	≥70 years	Total
Total (*N*, %)	3,277,247 (55.6)	1,832,912 (31.1)	779,282 (13.2)	5,889,441 (100)
Follow-up years (mean ± SD)	6.1 ± 2.1	7.1 ± 1.4	6.3 ± 2.1	6.4 ± 1.9
Age at baseline (mean ± SD)	33.0 ± 9.7	59.8 ± 5.6	78.3 ± 5.9	47.3 ± 18.9
Calendar year at baseline				
2009–2012	2,718,719 (83.0)	1,763,903 (96.2)	751,149 (96.4)	5,233,771 (88.9)
2013–2016	558,528 (17.0)	69,009 (3.8)	28,133 (3.6)	655,670 (11.1)
Sex				
Male	1,622,126 (49.5)	904,510 (49.3)	343,479 (44.1)	2,870,115 (48.7)
Female	1,655,121 (50.5)	928,402 (50.7)	435,803 (55.9)	3,019,326 (51.3)
Education level				
Low (≤9 years)	312,414 (9.5)	380,862 (20.8)	367,924 (47.2)	1,061,200 (18.0)
Medium (>9 and <12 years)	1,820,628 (55.6)	797,146 (43.5)	266,766 (34.2)	2,884,540 (49.0)
High (≥12 years)	1,119,148 (34.1)	554,112 (30.2)	138,763 (17.8)	1,812,023 (30.8)
Unknown	25,057 (0.8)	100,792 (5.5)	5829 (0.7)	131,678 (2.2)
Family income				
Low	1,083,681 (33.1)	509,576 (27.8)	235,620 (30.2)	1,828,877 (31.1)
Medium	1,029,300 (31.4)	614,210 (33.5)	297,145 (38.1)	1,940,655 (33.0)
High	1,164,266 (35.5)	709,126 (38.7)	246,517 (31.6)	2,119,909 (36.0)
Smoking-related diseases^a^				
No	3,261,572 (99.5)	1,798,045 (98.1)	744,499 (95.5)	5,804,116 (98.6)
Yes	15,675 (0.5)	34,867 (1.9)	34,783 (4.5)	85,325 (1.4)
Alcohol-related disorders and diseases^b^				
No	3,194,258 (97.5)	1,777,948 (97.0)	766,867 (98.4)	5,739,073 (97.4)
Yes	82,989 (2.5)	54,964 (3.0)	12,415 (1.6)	150,368 (2.6)
Family history of pancreatic cancer				
No	3,249,705 (99.2)	1,816,066 (99.1)	713,723 (91.6)	5,779,494 (98.1)
Yes	7537 (0.2)	12,542 (0.7)	3418 (0.4)	23,497 (0.4)
Unknown	20,005 (0.6)	4304 (0.2)	62,141 (8.0)	86,450 (1.5)
Dental health status^c^				
Healthy	1,729,541 (52.8)	791,990 (43.2)	352,698 (45.3)	2,874,229 (48.8)
Caries	570,397 (17.4)	271,550 (14.8)	120,480 (15.5)	962,427 (16.3)
Root canal infection	142,893 (4.4)	93,411 (5.1)	29,817 (3.8)	266,121 (4.5)
Mild inflammation	627,207 (19.1)	340,882 (18.6)	133,740 (17.2)	1,101,829 (18.7)
Periodontitis	207,209 (6.3)	335,079 (18.3)	142,547 (18.3)	684,835 (11.6)
Number of teeth				
Mean ± SD	29.0 ± 2.5	26.1 ± 4.6	20.8 ± 6.9	26.9 ± 5.0
28+	2,274,139 (69.4)	796,321 (43.4)	99,208 (12.7)	3,169,668 (53.8)
25–27	305,862 (9.3)	496,208 (27.1)	148,694 (19.1)	950,764 (16.1)
21–24	70,767 (2.2)	230,119 (12.6)	177,188 (22.7)	478,074 (8.1)
15–20	8343 (0.3)	95,266 (5.2)	153,240 (19.7)	256,849 (4.4)
1–14	8120 (0.2)	55,622 (3.0)	123,120 (15.8)	186,862 (3.2)
Unknown	610,016 (18.6)	159,376 (8.7)	77,832 (10.0)	847,224 (14.4)

*SD* standard deviation.

^a^Smoking-related diseases include chronic obstructive pulmonary disease and tobacco abuse (Supplementary Table 4).

^b^Alcohol-related disorders and diseases include vitamin B deficiency with alcoholism, alcoholic psychosis, alcoholism, alcohol-induced pseudo-Cushing syndrome, alcoholic neuropathy, alcoholic cardiomyopathy, gastritis, nervous system injury/disease, myopathy, liver cirrhosis, fatty liver, unspecified liver injury, liver fibrosis/sclerosis/failure, or pancreatitis due to alcohol, alcoholic hepatitis, care of pregnant mother with alcohol abuse in which the foetus may be affected, toxic effect of ethanol, high blood levels of alcohol, and alcohol abuse (Supplementary Table 4).

^c^The healthy group includes individuals without caries, root canal infection, mild inflammation, mild to advanced periodontitis, or periimplantitis; mild inflammation includes gingivitis, pericoronitis, stomatitis, mucositis, and other unspecified inflammatory conditions; periodontitis includes mild and advanced periodontitis, and periimplantitis.

A total of 10,081 pancreatic cancer cases were identified during follow-up. In our multivariable-adjusted model (Table 2), individuals <50 years with root canal infection had a 58% increased risk of pancreatic cancer than those with healthy dental status at baseline (multivariable-adjusted HR = 1.58, 95% CI 1.10–2.28), while individuals with mild dental inflammation and periodontitis had a 35% (95% CI 6–72%) and 56% (95% CI 17–108%) higher risk of pancreatic cancer, respectively, compared with the healthy reference (*P* for trend <0.001). In the 50–70 age group, only individuals with periodontitis had a 20% increased risk of pancreatic cancer (multivariable-adjusted HR = 1.20, 95% CI 1.11–1.29). Among individuals aged 70 years and older, HRs were around one (with CIs across one).

**Table 2:** Hazard ratios (HRs) and 95% confidence intervals (CIs) for pancreatic cancer according to dental health status at first visits, stratified by age at entry.

Groups	Person-years (*100,000)	Cancer cases (%)	Crude model^a^, HR (95% CI)	Multivariable-adjusted model^b^, HR (95% CI)
*>19 and <50 years*				
Total	197.1	464 (100)		
Healthy	104.3	190 (40.9)	Ref.	Ref.
Caries	35.5	78 (16.8)	1.09 (0.82–1.45)	1.13 (0.87–1.47)
Root canal infection	8.3	35 (7.5)	1.74 (1.14–2.64)*	1.58 (1.10–2.28)*
Mild inflammation	36.3	98 (21.1)	1.37 (1.06–1.77)*	1.35 (1.06–1.72)*
Periodontitis	12.7	63 (13.6)	1.68 (1.24–2.28)***	1.56 (1.17–2.08)**
*P* for trend^c^			<0.001	<0.001
*≥50 and <70 years*				
Total	128.9	4724 (100)		
Healthy	56.2	1942 (41.1)	Ref.	Ref.
Caries	19.1	654 (13.8)	1.00 (0.91–1.09)	0.98 (0.90–1.08)
Root canal infection	6.1	226 (4.8)	1.12 (0.97–1.28)	1.05 (0.92–1.21)
Mild inflammation	24.2	808 (17.1)	0.96 (0.88–1.43)	0.98 (0.90–1.06)
Periodontitis	23.3	1094 (23.2)	1.25 (1.16–1.35)***	1.20 (1.11–1.29)***
*P* for trend^c^			<0.001	<0.001
*≥70 years*				
Total	48.2	4893 (100)		
Healthy	21.6	2229 (45.6)	Ref.	Ref.
Caries	7.4	727 (14.9)	0.95 (0.87–1.03)	0.95 (0.87–1.03)
Root canal infection	1.8	179 (3.7)	0.98 (0.84–1.14)	0.98 (0.84–1.14)
Mild inflammation	8.5	838 (17.1)	0.96 (0.89–1.04)	0.97 (0.90–1.05)
Periodontitis	8.9	920 (18.8)	1.02 (0.94–1.10)	1.01 (0.94–1.10)
*P* for trend^c^			0.965	0.882

*P < 0.05; **P < 0.01; ***P < 0.001.

^a^Time scale is attained age, crude model.

^b^Time scale is attained age, with adjustment for sex, calendar year, family income, education, family history of pancreatic cancer, smoking-related diseases, alcohol-related disorders and diseases, and number of teeth.

^c^Cochran–Armitage test was used to test for trend.

We found that individuals with fewer teeth tended to have an increased risk of pancreatic cancer in all three age groups (Table 3). Additionally, individuals in the 50–70 age group with unknown number of teeth had a 33% increased risk of pancreatic cancer (multivariable-adjusted HR = 1.33, 95% CI 1.19–1.48) compared with those with full dentition. When number of teeth was treated as a time-varying exposure, the results remain similar as in the baseline models (Supplementary Table 7).

**Table 3:** Hazard ratios (HRs) and 95% confidence intervals (CIs) for pancreatic cancer according to number of teeth at first visits, stratified by age at entry.

Groups	Person-years (*100,000)	Cancer cases (%)	Crude model^a^, HR (95% CI)	Multivariable-adjusted model^b^, HR (95% CI)
*>19 and <50 years*				
Total	197.1	464 (100)		
28+	137.5	289 (62.3)	Ref.	Ref.
25–27	19.4	82 (17.7)	1.37 (1.07–1.76)*	1.28 (1.00–1.65)*
21–24	4.4	21 (4.5)	1.59 (1.02–2.48)*	1.41 (0.90–2.20)
1–20^c^	1.1	10 (2.2)	2.59 (1.38–4.87)**	2.09 (1.10–3.95)*
Unknown	34.6	62 (13.4)	1.33 (1.01–1.75)*	1.22 (0.93–1.62)
*P* for trend^d^			<0.001	0.004
*≥50 and <70 years*				
Total	128.9	4724 (100)		
28+	56.1	1492 (31.6)	Ref.	Ref.
25–27	35.7	1317 (27.9)	1.12 (1.04–1.20)**	1.11 (1.03–1.19)**
21–24	16.4	795 (16.8)	1.25 (1.15–1.37)***	1.21 (1.11–1.33)***
15–20	6.6	416 (8.8)	1.47 (1.32–1.64)***	1.38 (1.23–1.55)***
1–14	3.7	262 (5.5)	1.64 (1.44–1.80)***	1.51 (1.32–1.73)***
Unknown	10.5	442 (9.4)	1.40 (1.26–1.56)***	1.33 (1.19–1.48)***
*P* for trend^d^			<0.001	<0.001
*≥70 years*				
Total	48.2	4893 (100)		
28+	6.3	591 (12.1)	Ref.	Ref.
25–27	9.9	939 (19.2)	1.01 (0.91–1.12)	1.02 (0.92–1.13)
21–24	11.5	1156 (23.6)	1.04 (0.94–1.15)	1.04 (0.94–1.15)
15–20	9.5	947 (19.4)	1.01 (0.91–1.12)	1.00 (0.90–1.11)
1–14	7	819 (16.7)	1.15 (1.03–1.28)**	1.13 (1.01–1.26)*
Unknown	10.5	441 (9.0)	1.10 (0.97–1.24)	1.07 (0.94–1.21)
*P* for trend^d^			0.016	0.049

*P < 0.05; **P < 0.01; ***P < 0.001.

^a^Time scale is attained age, crude model.

^b^Time scale is attained age, with adjustment for sex, calendar year, family income, education, family history of pancreatic cancer, smoking-related diseases, alcohol-related disorders and diseases, and dental health status.

^c^In the <50 age group, the subgroups having 15–20 and 1–14 teeth were combined into 1–20 teeth subgroup due to the small number of cancer cases.

^d^Cochran–Armitage test was used to test for trend; the unknown category was not included for trend test.

**Fig. 2:**
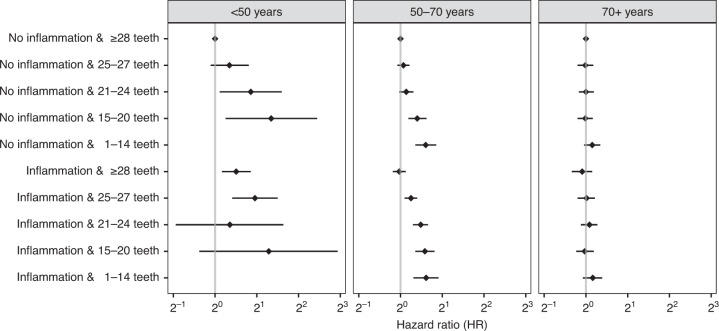
Multivariable-adjusted hazard ratios (HRs) for pancreatic cancer in relation to a combination of dental inflammatory conditions and number of teeth by age group. Time scale is attained age. The Cox models were adjusted for sex, calendar year, family income, education, family history of pancreatic cancer, smoking-related diseases, and alcohol-related disorders and diseases. Dental inflammation includes mild inflammation and periodontitis; no inflammation includes healthy, caries, and root canal infection; no inflammation and ≥28 teeth was the reference group; in the <50 age group, the subgroups having 15–20 and 1–14 teeth were combined into 1–20 teeth due to the small numbers of cancer cases, with the label of “(No) inflammation & 15–20 teeth”. *X*-axis is logged. *P* value for interaction was 0.469 in the <50 age group, 0.477 in the 50–70 age group, and 0.772 in the 70+ age group.

There were no interaction effects between dental inflammatory conditions and the number of teeth in all age groups with statistical significance (Fig. 2).

In the sensitivity analyses, when dental health status was treated as a time-varying exposure, an 8% increased cancer risk (crude HR = 1.08, 95% CI 1.01–1.17) in the ≥70 age group was observed in individuals with periodontitis than the healthy reference (Supplementary Table 7).

After adding a one-year lag since baseline, the HRs were similar for both dental health status and the number of teeth (data not shown). When the outcome was limited to incident pancreatic cancer cases only, the results changed marginally compared with the main analyses (data not shown).

## Discussion

In this nationwide Swedish registry-based cohort study in Sweden, we confirm that periodontitis increases the risk of pancreatic cancer by 20–56% in adults under 70 years as compared with the dentally healthy individuals. Our results also provide new evidence that root canal infection appears to increase the risk of pancreatic cancer, with risk in individuals younger than 50 years increased by up to 58%, similar to periodontitis. To our knowledge, this fact has not been reported previously. Similarly, individuals with fewer teeth tended to have an excess risk of pancreatic cancer.

Accumulating evidence supports periodontal pathogens are strongly linked to pancreatic cancer. As previously shown by Michaud and colleagues [22], individuals with a high level of antibodies against *Porphyromonas gingivalis* (>200 ng/ml) had a twofold higher risk of pancreatic cancer than individuals with a low level of the antibodies (≤200 ng/ml). Fan et al. [23] also found that *Aggregatibacter actinomycetemcomitans* was associated with a higher risk of pancreatic cancer (odds ratio [OR] = 2.20, 95% CI 1.16–4.18); in contrast, phylum *Fusobacteria* seemed to be associated with a decreased pancreatic cancer risk (OR = 0.94, 95% CI 0.89–0.99).

The keystone pathogen hypothesis suggests that *Aggregatibacter actinomycetemcomitans* and *Porphyromonas gingivalis*, may affect the inflammatory disease by proliferating, remodelling and controlling commensal microbiome [19]. Oral commensals, on the other hand, do cause root canal infection, with cell necrosis and tissue destruction involving the supportive tissues through the root canal that leads to apical periodontitis which in fact demonstrates a very similar periodontal pathogen profile [24].

Besides triggering systemic inflammation, the pathogenic species may promote the production of nitrosamines, which are potential carcinogens [25], and the release of enzymes and other carcinogenic metabolites [26]. Notably, the dissemination of these products can take place by several routes: (a) by entering the circulatory system via the perturbed periodontal tissues (oral–blood axis), (b) through the oral–gut axis via saliva, or (c) via the lymphatic draining system, possibly also attributing to immune modulations in distant organs [27–29].

We observed a positive association between root canal infection and pancreatic cancer risk among younger adults. Although this finding is new, it is not completely surprising: root canal infection often involves the apical periodontal tissues around the root—clinically, the infection can persist for multiple years without any symptoms [8]. Notably, oral bacteria have been found in cystic precursors to pancreatic cancer [30], but such precursor lesions could take up to ten years to become invasive. Although less explored, studies report that chronic apical periodontitis is associated with diabetes or cardiovascular diseases [31, 32]. Translocated root canal microbiomes, either microbial DNA or live bacteria, are found in distant organs including thrombus aspirates of subjects with myocardial infarction [33]. Future studies are warranted to confirm our findings.

We performed stratified analysis by age, since the effects of periodontal diseases accumulate with age. Compared with younger patients, uncontrolled lifetime periodontal diseases may have a greater impact on older patients [18]. However, an increased risk was observed between periodontitis and pancreatic cancer risk among individuals aged younger than 70 years, but not in 70 years and older. Some studies have previously reported that for dental pathology, many older adult patients may often be left untreated [18] due to overall health status. Therefore, it is possible that we grouped some older patients, who had compromised dental health but with no diagnosis/treatment records of periodontal diseases, into the dentally “healthy” subgroup. This misclassification is likely to be limited to younger individuals.

Tooth loss is a robust indicator of oral health, reflecting lifetime accumulation of oral inflammation. In this study, we demonstrated that individuals with fewer teeth had an excess risk of pancreatic cancer. Several studies reported null or non-statistically significant positive results about the association between the number of teeth and pancreatic cancer risk [6, 34, 35], and only one study in Finland reported a 63% increased risk in male smokers with complete edentulism (lack of teeth) with statistical significance (HR = 1.63, 95% CI 1.09–2.46) [36]. Inconsistent results are partly due to heterogeneity of the study populations, exposure ascertainment, confounding adjustment, and follow-up time.

Compared with prior studies, the strengths of this study are the large unselected population, and the detailed clinical information about exposures, although some subjects with financial distress may not seek dental health care when needed, due to partial out-of-pocket payment [16]. The study population consisted of subjects aged >19 years with at least one dental visit and data on oral health diagnoses and dental procedures in Sweden. The exposures of compromised dental health used in this study were defined by diagnostic codes from the Dental Health Register (uniquely used in Sweden) [17], which indicates that the probability of false positives is low. Another exposure, the number of teeth, which was captured from clinical examinations, is more accurate than self-reported data. In addition, the linkage to Swedish national registries [37, 38] allowed us to control for confounding factors, such as socioeconomic status and some diseases. With regard to diabetes, longitudinal studies indicate a bidirectional relationship between periodontitis and diabetes—that is, periodontitis worsens diabetes by increasing the inflammatory burden and enhancing insulin resistance, and vice versa [9, 11]. Diabetes is a risk factor as well as a consequence of a tumour in the pancreas. Therefore, we did not adjust for diabetes in the Cox models. Moreover, we added a lag time (one year) after the start of follow-up to minimise the potential risk of reverse causality or surveillance bias. It turned out that HRs were only marginally changed when excluding the first year of follow-up, which indicates a minimal impact from such bias. Furthermore, when compared with general health care that addresses broad health conditions, dental care is limited to a much narrower field of health care: oral diseases and concerns. Therefore, the surveillance bias is likely to be limited.

The current study has some limitations. First, the longest follow-up period in this cohort is eight years, thus we probably could not capture all potential cancer cases, as carcinogenesis from initiation to metastasis can last more than ten years [39]. However, the number of teeth reflects the accumulation of oral inflammation over a lifetime, so we may be able to study late-occurring outcomes without an extended follow-up period. Another caveat to consider is the definition of dental health conditions: since most oral-related diseases and concerns were dealt with in public and private dental clinics, and were assessed by many different dentists, inter- and intra-observer variation is a reality. In addition, the registries did not contain data on lifestyle factors and medications, such as smoking, alcohol drinking, antibiotic or anti-inflammatory treatment, thus we could not adjust for these variables directly; though we used proxies for smoking and alcohol drinking during analyses, residual confounding cannot be ruled out. Having fewer teeth reduces masticatory ability, which may result in a less healthy diet [40, 41]; a diet with fewer vegetables and fibres and more cholesterol and calories, increases the general risk of cancer [41]. In the current study, information about dietary habits was not available from any registry and was thus not included in the models. Nevertheless, other studies have adjusted for covariates related to diet when conducting multivariable models, but the results changed little [34, 42, 43]. At last, this is a Swedish population-based cohort and has limitations for generalisation. Other potential biases with minimal impact on risk are described in the Supplementary Discussion.

In conclusion, this study offers supporting evidence that periodontal disease and tooth loss are associated with increased risks of pancreatic cancer. The positive association between root canal infection and pancreatic cancer risk among individuals younger than 50 years is a new finding. Further prospective studies that identify specific oral pathogenic bacteria which precede carcinoma development are warranted, and these findings may also provide clues for non-invasive biomarkers that can be used to identify tumours at an early stage in high-risk populations.

## Supplementary information

Supplementary material

## Data Availability

The data that support the findings of this study are not publicly available due to privacy and ethical restrictions. The data are available from the corresponding author WY on reasonable request.

## Code availability

Scripts used to generate the results presented in this study are available from the corresponding author JY on reasonable request.
