# Supplementary material for: Poor dental health and risk of pancreatic cancer: a nationwide registry-based cohort study in Sweden, 2009–2016
Source: Br J Cancer. 2022 Oct 22;127(12):2133–40. doi: 10.1038/s41416-022-02018-8 (PMC9726876; doi:10.1038/s41416-022-02018-8)
Supplement: Supplementary file 1 — Supplementary material [file 41416_2022_2018_MOESM1_ESM.pdf]

Supplementary material

Poor dental health and risk of pancreatic cancer: A nationwide registry-based cohort study in Sweden, 2009-2016

Jingru Yu*, Alexander Ploner, Margaret Sällberg Chen, Ji Zhang, Gunilla Sandborgh-Englund, Weimin Ye*

*, corresponding authors.

Supplementary Methods

The Swedish Dental Health Register (DHR)

The DHR has been described elsewhere [1]. Briefly, the DHR was initiated in July 2008, with good coverage from 2009, containing subjects' dental care information from both public and private dental care organizations in Sweden. This registry has high coverage of the Swedish adult population, with about 75% of individuals >19 years visiting dental clinics on a regular basis [2]. The DHR records clinical and administrative data, including number of teeth, diagnoses, dental procedures, and visit dates. However, only diagnoses that are subject to an intervention or treatment during the dental visit are recorded [1]. In general, an oral health examination is performed at a subject's first visit; if needed, further treatment will be performed in the following months. Thus, we used information of diagnoses and dental procedures from the first dental visit and all following visits within three months to establish a baseline diagnosis.

Exposures

For dental health status, when multiple diagnoses were present within the same date, the most severe according to the order of increasing severity was selected. Consequently, the most severe diagnosis across the first visit up until three months was ascertained as the baseline dental health status, and the date of first dental visit was set as date of entry into the cohort.

In addition, the information of number of teeth was matched to dental care data by the visiting dates. If a same-date record could not be found, then the information from the closest record up to six months before or five years after the first dental visit (entry date) was used. Additionally, records reported zero or 32 remaining and intact teeth were excluded, since we considered this type of record as unacceptably low quality based on the results in a validation study of the DHR [1].

Interactions for preliminary models

We estimated the interactions between age at baseline and poor dental health, and between age at baseline and number of teeth. To consider an interaction term, we multiplied the two terms together (continuous age*categorized dental health status / number of teeth), and compared the main effect model (not including the interaction term) with the interaction model (including the interaction term). A likelihood ratio test was used to assess the null hypothesis of no interaction.

Supplementary Discussion

Selection bias ─ Selection bias due to loss to follow-up is caused by the differences in completeness of follow-up between exposed and non-exposed groups, which is a common concern in cohort studies. In this study, we linked multiple nationwide registers with almost

complete information from birth to death (i.e., migration, death, and outcome of interest), to minimize chances of loss to follow-up for each enrolled subject.

Reverse causality ─ Due to a lack of efficient diagnostic tools for pancreatic cancer, we were not able to identify early-stage pancreatic cancer or precursor lesions as the outcome of interest in the cohort. It implies that the observed exposure-outcome association is biased, due to the fact that the outcome may already exist before exposure occurs. Therefore, we added a lag time (one year) after the start of follow-up to minimize the risk of such bias. However, hazard ratios (HRs) were only marginally changed, which indicates a minimal impact from reverse causality.

Misclassification ─ It is possible that incident pancreatic cancer is underreported due to current diagnostic techniques and patient register system. In this study, pancreatic cancer cases were ascertained from both the cancer (incident cases) and cause of death registers (death cases) to estimate relative risks. HRs changed marginally when comparing the estimates based on incident pancreatic cancers only, with those combining incident and death cases, which indicates that underreporting of incident cancers does not strongly bias the results.

Surveillance bias ─ It may occur when some participants are followed up more closely and receive more surveillance, screening or tests than others, leading to an outcome diagnosed more frequently in these closely investigated individuals [3]. The general health care addresses broad health conditions and focuses on some specific areas of health if necessary, while dental care is limited to narrower field of health care: oral diseases and concerns. In this study, the relative risk estimates was only marginally changed when excluding the first year of follow-up, which indicates a minimal impact from surveillance bias. Therefore, this bias is likely to be limited.

Supplementary Figure

Supplementary Figure. Directed Acyclic Graph (DAG) for this cohort study

Supplementary Tables

Supplementary Table 1. Characteristics of previous important studies about the association between poor dental health and risk of pancreatic cancer

Supplementary Table 2. Diagnostic codes for dental health status

Supplementary Table 3. Cut-off values for the tertiles of the family disposable income from the Swedish general population aged 20-65 years and older than 65 years, 2008-2015

Supplementary Table 4. ICD codes for smoking-related diseases, and alcohol-related disorders and diseases

Supplementary Table 5. Characteristics of all participants at first visits by dental health status, in the cohort identified from the Swedish Dental Health Register, 2009-2016

Supplementary Table 6. Characteristics of all participants at first visits by number of teeth, in the cohort identified from the Swedish Dental Health Register, 2009-2016

Supplementary Table 7. Time-varying hazard ratios (HRs) with 95% confidence intervals (CIs) for pancreatic cancer according to dental health status and number of teeth, stratified by age at entry, in the cohort identified from the Swedish Dental Health Register, 2009-2016

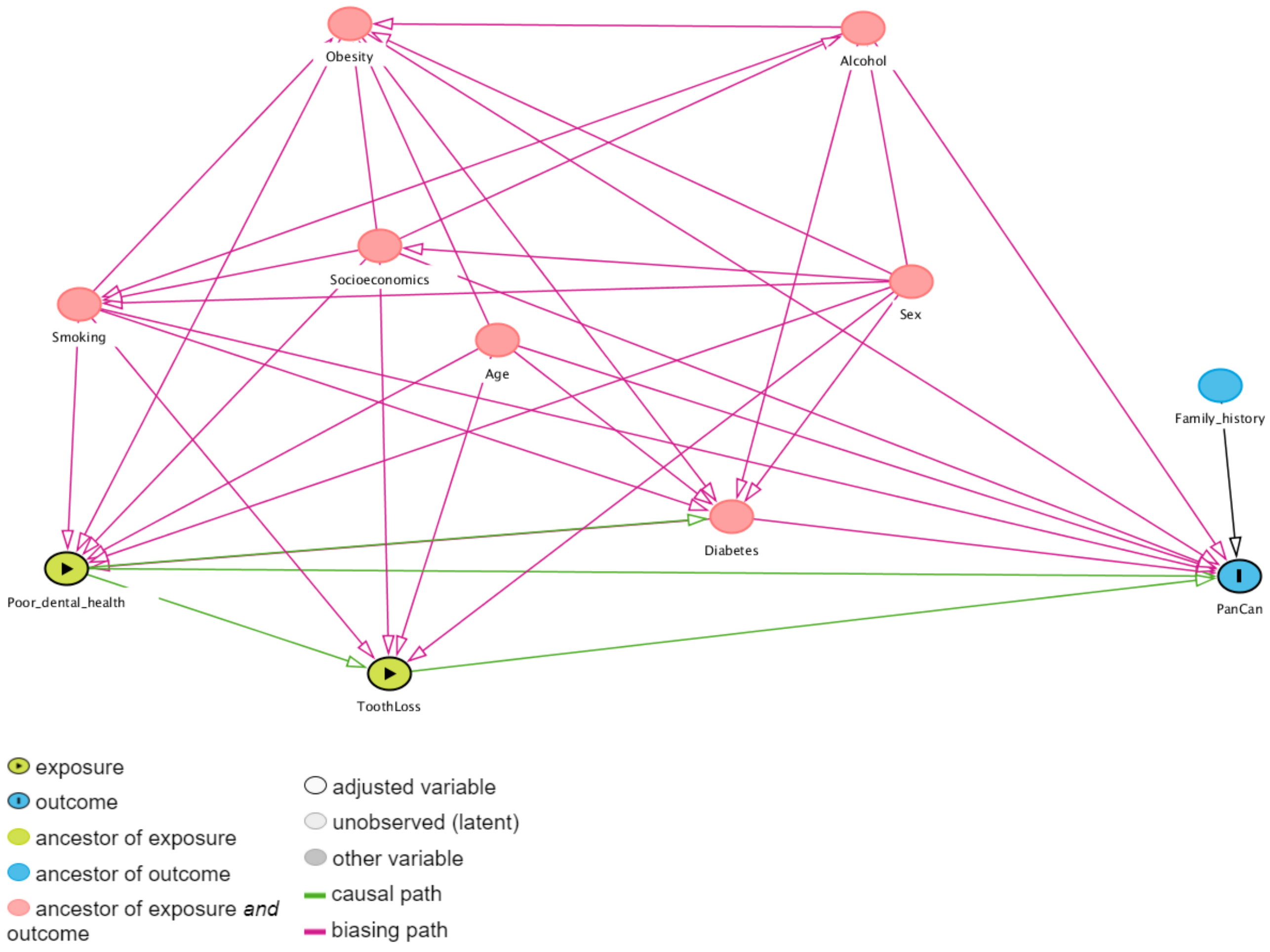

Supplementary Figure. Directed Acyclic Graph (DAG) for this cohort study (The number of teeth [ToothLoss] was adjusted besides covariates mentioned in the DAG when estimating risk effects of poor dental health [Poor_dental_health], and poor dental health status was also adjusted for estimating risk effects of number of teeth.)

Supplementary Table 1. Characteristics of previous important studies about the association between poor dental health and risk of pancreatic cancer

Studies	Study periods	No. of subjects (case/control or event/cohort participants)	Dental health-related factors (exposure measurement)	Risk estimates (95% CI)	Adjustments
Incidence (n=16)					
Cohort: Stolzenberg-Solomon, 2003 [4]	1985-1997, Finland	174 events/29,104 male smokers	**Missing teeth** (self-reported)<br>0-10<br>11-31<br>Edentulous	 <br>Ref.<br>HR = 1.23 (0.82-1.85)<br>HR = 1.63 (1.09-2.46)	Age, number of years of smoking, education, urban living, and height
Cohort: Michaud, 2007 [5][a]	1986-2002, USA	216 events/48,375 men	**Periodontal disease** (self-reported)<br>No<br>Yes	 <br>Ref.<br>RR = 1.64 (1.19-2.26)	Age, smoking history, profession, race, geographic location, physical activity, diabetes, BMI, height, cholecystectomy, NSAID use, multivitamin use, baseline teeth number, dietary factors, and total calories
Cohort: Michaud, 2008 [6]	1986-2004, USA	253 events/48,375 men	**Periodontal disease** (self-reported)<br>No<br>Yes<br>**Number of teeth**<br>25-32<br>17-24<br>0-16	 <br>Ref.<br>HR = 1.54 (1.16-2.04)<br><br>Ref.<br>HR = 1.08 (0.77-1.52)<br>HR = 0.91 (0.56-1.47)	Age, ethnic origin, physical activity, history of diabetes, alcohol, BMI, geographical location, height, calcium intake, total caloric intake, red-meat intake, fruit and vegetable intake, vitamin D score, and smoking
Case-control: Hiraki, 2008 [7]	2001-2005, Japan	178 cases/10,480 controls	**Remaining teeth** (self-reported)<br>21+<br>9-20<br>1-8<br>0	 <br>Ref.<br>OR = 1.33 (0.86-2.07)<br>OR = 0.60 (0.32-1.14)<br>OR = 1.33 (0.57-3.10)	Age, sex, smoking and drinking status, vegetable and fruit intake, BMI, and regular exercise
Cohort: Arora, 2010 [8]	1963-2004, Sweden	142 events/15,333 Swedish twins	**Periodontal disease** (self-reported)<br>No<br>Yes	 <br>Ref.<br>HR = 2.06 (1.14-3.75)	Sex, age, education, employment, number of siblings, smoking status (or of partner), alcohol status, diabetes, and BMI

Nested case-control: Michaud, 2013 [9]	1992-2000, Europe	405 cases/416 controls	**Plasma *Porphyromonas gingivalis***<br>Low antibody levels<br>High antibody levels<br>**Commensal oral bacteria**<br>Low antibody levels<br>High antibody levels	 Ref.<br>OR = 2.14 (1.05-4.36)<br><br>Ref.<br>OR = 0.55 (0.36-0.83)	Centre, sex, follow-up time, age at blood collection, date, and time of blood collection, fasting status and use of exogenous hormones among women, BMI, and smoking status
Cohort: Wen, 2014 [10]	1997-2010, Taiwan	84 events/148,166 participants	**Periodontal disease**<br>(health claim records)<br>Gingivitis<br>Periodontitis	 <br>Ref.<br>HR = 1.15 (0.75-1.78)	Sex, age, and the presence of comorbidities
Cohort: Hwang, 2014 [11]	1997-2010, Taiwan	102 events/116,706 participants	**Periodontal disease**<br>(health claim records)<br>Not Treated<br>Treated	 <br>Ref.<br>HR = 0.55 (0.35, 0.85)	Age, sex, occupation, type 2 diabetes mellitus, hypertension, and hyperlipidemia
Cohort: Chang, 2016 [12]	1998-2005, Taiwan	107 events/214,890 participants	**Periodontal disease**<br>(health claim records)<br>No<br>Gingivitis or periodontitis<br>Other PD<br>Any PD	 <br>Ref.<br>HR = 1.67 (1.05-2.66)<br>HR = 1.42 (0.87-2.30)<br>HR = 1.55 (1.02-2.33)	Age, sex, diabetes, hyperlipidemia, allergies, viral hepatitis, peptic ulcer, pancreatitis, COPD, and alcohol-related conditions
Cohort: Huang, 2016 [13]	1973-1974, follow-up to 2012, Sweden	126 events/19,924 participants	**Remaining teeth**<br>(dental examination)<br>21-32<br>11-20<br>0-10<br>**Dental plaque**<br>No<br>Acceptable<br>Unacceptable	 <br>Ref.<br>HR = 1.2 (0.7-2.0)<br>HR = 1.3 (0.7-2.3)<br><br>Ref.<br>HR = 1.8 (0.9-3.6)<br>HR = 2.1 (1.0-4.7)	Age, sex, attained calendar period, tobacco use, alcohol consumption, and area of residence
Cohort: Michaud, 2016 [14]	1986-2012, USA	141 events/19,933 male non-smokers	**Periodontal disease**<br>(Self-reported)<br>No<br>Yes<br>**Number of teeth**<br>25-32 teeth<br>17-24 teeth<br>0-16 teeth	 <br>Ref.<br>RR = 1.57 (0.98-2.50)<br><br>Ref.<br>HR = 0.92 (0.52-1.61)<br>HR = 1.10 (0.48-2.51)	Age, race, alcohol use, physical activity, history of diabetes, BMI, geographical location, height, and NSAID use

Cohort: Nwizu, 2017 [15]	1999-2003, follow-up to 2013	272 events/ 65,869 women	**Periodontal disease** (Self-reported)<br>No<br>Yes	 <br>Ref.<br>HR = 0.89 (0.67-1.18)	Age, pack-years, and BMI
Nested case-control: Fan, 2018 [16]	1992-2008 (cohort 1), 1993-2010 (cohort 2), USA	361 cases/371 controls	**Oral pathogen, *Porphyromonas gingivalis***<br>Absence<br>Presence<br>***Aggregatibacter actinomycetemcomitans***<br>Absence<br>Presence	 Ref.<br>OR = 1.60 (1.15-2.22)<br><br><br><br>Ref.<br>OR = 2.20 (11.16-4.18)	Random effect of cohorts, age, race, sex, BMI, smoking status, alcohol consumption, and history of diabetes
Cohort: Michaud, 2018 [17]	1987-1989, follow-up to 1996-1998, USA	48 events/1,627 never smoking ARIC participants	**Periodontitis** (dental examination)<br>No/Mild<br>Severe	 <br>Ref.<br>HR = 1.65 (0.72-3.77)	Age, field center, education level, drinking status, BMI, diabetes status, joint terms for sex and HRT use, and joint terms for field center and race
Cohort: Jordão, 2019 [18]	2006-2010, follow-up to 2014, UK	436 events/465,637 participants	**Oral health** (self-reported)<br>Good (month ulcers, toothache, dentures)<br>Poor (painful, bleeding gums, and loose teeth)	 <br>Ref.<br><br>HR = 1.05 (0.80-1.39)	Age, sex, socioeconomic status, tertiary education degree, smoking status by pack years, alcohol status, BMI, waist circumference, and daily consumption of fruit and vegetables
Cohort: Lee, 2020 [19]	2002-2015, Korea	1,579 events/150,774 participants	**Periodontal disease** (health claims data)<br>No<br>Yes<br>**Number of missing teeth**<br>0<br>1-7<br>8-14<br>15+	 <br>Ref.<br>HR = 0.98 (0.85-1.14)<br><br>Ref.<br>HR = 1.00 (0.88-1.12)<br>HR = 1.03 (0.74-1.44)<br>HR = 0.98 (0.67-1.42)	age, sex, income level, alcohol consumption, smoking status, regular exercise, hypertension, BMI, diabetes, dyslipidemia, renal diseases, systolic blood pressure, total cholesterol, fasting blood sugar, aspartate aminotransferase, alanine aminotransferase, gamma glutamyl transpeptidase, proteinuria, periodontal disease, frequency of toothbrushing, dental visit for any reason, professional dental cleaning and number of missing teeth
Cohort: Zhang, 2022 [20]	2004-2015, China	585 events/510,148 participants	**Oral health** (self-reported)<br>Good (gums bleed when brushing teeth: never,	 <br>Ref.	Age, sex, BMI, study sites, education level, marital status, household income per year, alcohol consumption, smoking status, physical activity, aspirin prescription for CVD, menopausal status,

			occasionally, or sometimes<br>Poor (gums bleed when brushing teeth: always, or rarely or never brushing teeth)	 <br>HR = 0.94 (0.76-1.17)	personal history of diabetes, and family history of cancer
Mortality (n=5)					
Cohort: Hujoel, 2003 [21]	1971-1975, follow-up to 1992, USA	49 deaths/11,328 participants	**Periodontal status (**dental examination**)**<br>Healthy Periodontium<br>Gingivitis<br>Periodontitis<br>Edentulism	 <br>Ref.<br>OR=1.44 (0.67-3.11)<br>OR=1.77 (0.85-3.67)<br>OR=1.90 (0.95-3.81)	Age, sex
Cohort: Ahn, 2012 [22]	1988-1994, follow-up to 2004, USA	18 deaths/12,605 participants	**Periodontal disease**<br>(dental examination)<br>No<br>Yes	 <br>Ref.<br>RR = 4.56 (0.93-22.3)	Age, sex, smoking, education, race/ethnicity, and BMI
Cohort: Ansai, 2013 [23]	1998-2010, Japan	4 deaths/656 elderly participants (≥80 years)	**Missing teeth number (**dental examination, continuous variable**)**	HR = 0.96 (0.83-1.11)	Sex and smoking status, total cholesterol, serum albumin, fasting serum glucose, BMI, physical activity, and place of residence
Cohort: Heikkilä, 2018 [24]	2001-2002, follow-up to 2013, Finland	75 deaths/68,273 participants	**Periodontitis**<br>(dental care data)<br>No<br>Yes	 <br>Ref.<br>MRR = 2.28 (1.31-3.98)	Calendar time, age, sex, socioeconomic status, number of teeth, dental treatments (gingivitis, caries, endodontic caries, surgery and prosthesis), oral health indices, need of periodontal treatment, and diabetes
Cohort: Chung, 2020 [25]	2005-2008, follow-up to 2012	208 deaths/82,548 elderly participants (≥65 years)	**Periodontal disease**<br>(dental examination)<br>No<br>Yes	 <br>Ref.<br>HR = 1.02 (0.79-1.31)	Age, sex
Cohort: Zhang, 2022 [20]	2004-2015, China	409 events/510,148 participants	**Oral health**<br>(self-reported)<br>Good (gums bleed when brushing teeth: never, occasionally, or sometimes<br>Poor (gums bleed when brushing teeth: always, or rarely or never brushing teeth)	 <br>Ref.<br><br><br><br>HR = 0.94 (0.73-1.21)	Age, sex, BMI, study sites, education level, marital status, household income per year, alcohol consumption, smoking status, physical activity, aspirin prescription for CVD, menopausal status, personal history of diabetes, and family history of cancer

PD, periodontal disease; CI, confidence interval; RR, relative risk; OR, odds ratio; HR, hazard ratio; MRR, mortality rate ratio; BMI, body mass index; NSAID, non-steroidal anti-inflammatory drug; COPD, chronic obstructive pulmonary disease.

[a]Participants in two studies overlapped; the results were included in the study from Michaud et al., 2008 for the pancreatic cancer analysis.

Supplementary Table 2. Diagnostic codes for dental health status#

Categories	Subgroups	Diagnostic codes
4	Periodontitis	3043: Periodontitis
		3044: Periimplantiits
3	Mild inflammation	3042: Mucositis (implants)
		3045: Pericoronitis
		3041: Gingivitis
		3046: Other unspecific inflammation conditions
		3072, 3073: Stomatitis
2	Root canal infection	3051: Root canal infection and treatment
1	Caries	4001, 4002, 4011, 4012: Caries
0	Healthy	

#These codes are specific in the Swedish Dental Health Register (DHR) [26].

Supplementary Table 3. Cut-off values for the tertiles of the family disposable income from the Swedish general population aged 20-65 years and older than 65 years, 2008-2015#

Year	20-65 years		>65 years	
	**33% cut-off in 1000 SEK**	**67% cut-off in 1000 SEK**	**33% cut-off in 1000 SEK**	**67% cut-off in 1000 SEK**
2008	255	467	148	262
2009	259	481	154	273
2010	267	499	156	279
2011	278	520	165	295
2012	285	538	175	312
2013	289	548	182	325
2014	299	570	184	332
2015	309	591	188	342

#In the 20-65 age group, the average ranges of low, medium, and high level are <280,000 SEK, 280,000-520,000 SEK, and >520,000 SEK, respectively; in the >65 age group, the average ranges of low, medium, and high level are <169,000 SEK, 169,000-303,000 SEK, and >303,000 SEK, respectively.

Supplementary Table 4. ICD codes for smoking-related diseases, and alcohol-related disorders and diseases

Parameters	ICD-7	ICD-8	ICD-9	ICD-10
Smoking-related diseases				
Tobacco abuse	-	989.9	305B	Z71.6, Z72.0, F17, T65.2, Z864A
COPD	501, 502	490-492	490-492, 496	J40-J44
Alcohol-related disorders and diseases [27,28]*				
	280.00, 281.00, 307, 322, 581.10, 583.10, 960.20, 960.29	261.00, 262.00, 291, 303, 571.00, 571.01, 980.00, 980.01, 980.99	291, 303, 305A, 357F, 425F, 535D, 571A, 571B, 571C, 571D, 980A, 980X, 790D, 977D, V79B	E24.4, F10, G62.1, I42.6, K29.2, G31.2, G72.1, K70.0, K70.1, K70.2, K70.3, K70.4, K70.9, K85.2, K86.0, O35.4, T51.0, T51.9, R78.0, Y57.3, X65, Y90, Y91, Z50.2, Z71.4, Z72.1

ICD, International Classification of Diseases; COPD, chronic obstructive pulmonary disease.
*Alcohol-related disorders and diseases include vitamin B deficiency with alcoholism, alcoholic psychosis, alcoholism, alcohol-induced pseudo-Cushing syndrome, alcoholic neuropathy, alcoholic cardiomyopathy, gastritis, nervous system injury/disease, myopathy, liver cirrhosis, fatty liver, unspecified liver injury, liver fibrosis/sclerosis/failure, or pancreatitis due to alcohol, alcoholic hepatitis, care of pregnant mother with alcohol abuse in which the fetus may be affected, toxic effect of ethanol, high blood levels of alcohol, and alcohol abuse.

Supplementary Table 5. Characteristics of all participants at first dental visits by dental health status, in the cohort identified from the Swedish Dental Health Register, 2009-2016

**Characteristics**	**Dental health status (number of individuals, %)**					
	**Healthy**	**Caries**	**Root canal infection**	**Mild inflammation**[a]	**Periodontitis**[b]	**Total**
Total	2,874,229 (48.8)	962,427 (16.3)	266,121 (4.5)	1,101,829 (18.7)	684,835 (11.6)	5,889,441 (100)
Age at baseline						
Mean ± SD	45.2 ± 19.3	46.6 ± 18.4	49.1 ± 16.5	46.9 ± 18.4	57.3 ± 15.8	47.3 ± 18.9
>19 and <50	1,729,541 (60.2)	570,397 (59.3)	142,893 (53.7)	627,207 (56.9)	207,209 (30.3)	3,277,247 (55.6)
≥50 and <70	791,990 (27.6)	271,550 (28.2)	93,411 (35.1)	340,882 (30.9)	335,079 (48.9)	1,832,912 (31.1)
≥70	352,698 (12.3)	120,480 (12.5)	29,817 (11.2)	133,740 (12.1)	142,547 (20.8)	779,282 (13.2)
Calendar year of baseline						
2009-2012	2,557,022 (89.0)	873,116 (90.7)	224,895 (84.5)	951,723 (86.4)	627,015 (91.6)	5,233,771 (88.9)
2013-2016	317,207 (11.0)	89,311 (9.3)	41,226 (15.5)	150,106 (13.6)	57,820 (8.4)	655,670 (11.1)
Sex						
Male	1,332,142 (46.3)	485,962 (50.5)	146,859 (55.2)	551,265 (50.0)	353,887 (51.7)	2,870,115 (48.7)
Female	1,542,087 (53.7)	476,465 (49.5)	119,262 (44.8)	550,564 (50.0)	330,948 (48.3)	3,019,326 (51.3)
Education level						
Low	469,920 (16.3)	178,799 (18.6)	63,731 (23.9)	178,357 (16.2)	170,393 (24.9)	1,061,200 (18.0)
Medium	1,433,747 (49.9)	477,974 (49.7)	135,986 (51.1)	522,068 (47.4)	314,765 (46.0)	2,884,540 (49.0)
High	913,238 (31.8)	286,761 (29.8)	60,153 (22.6)	376,490 (34.2)	175,381 (25.6)	1,812,023 (30.8)
Unknown	57,324 (2.0)	18,893 (2.0)	6,251 (2.3)	24,914 (2.3)	24,296 (3.5)	131,678 (2.2)
Family income						
Low	868,284 (30.2)	314,453 (32.7)	103,287 (38.8)	326,349 (29.6)	216,504 (31.6)	1,828,877 (31.1)
Medium	920,332 (32.0)	332,125 (34.5)	90,806 (34.1)	357,995 (32.5)	239,397 (35.0)	1,940,655 (33.0)
High	1,085,613 (37.8)	315,849 (32.8)	72,028 (27.1)	417,485 (37.9)	228,934 (33.4)	2,119,909 (36.0)
Smoking-related diseases						
No	2,836,589 (98.7)	949,436 (98.7)	260,888 (98.0)	1,088,832 (98.8)	668,371 (97.6)	5,804,116 (98.6)
Yes	37,640 (1.3)	12,991 (1.3)	5,233 (2.0)	12,997 (1.2)	16,464 (2.4)	85,325 (1.4)
Drinking-related disorders and diseases						
No	2,811,957 (97.8)	936,787 (97.3)	251,430 (94.5)	1,077,737 (97.8)	661,162 (96.5)	5,739,073 (97.4)
Yes	62,272 (2.2)	25,640 (2.7)	14,691 (5.5)	24,092 (2.2)	23,673 (3.5)	150,368 (2.6)
Family history of pancreatic cancer						
No	2,823,808 (98.2)	944,680 (98.2)	261,571 (98.3)	1,081,050 (98.1)	668,385 (97.6)	5,779,494 (98.1)
Yes	10,604 (0.4)	3,654 (0.4)	1,100 (0.4)	4,421 (0.4)	3,718 (0.5)	23,497 (0.4)
Unknown	39,817 (1.4)	14,093 (1.5)	3,450 (1.3)	16,358 (1.5)	12,732 (1.9)	86,450 (1.5)
Number of teeth						
Mean ± SD	27.1 ± 4.9	27.2 ± 4.8	26.7 ± 5.1	27.3 ± 4.4	25.3 ± 5.7	26.9 ± 5.0
28+	1,592,690 (55.4)	544,755 (56.6)	117,898 (44.3)	634,980 (57.6)	279,345 (40.8)	3,169,668 (53.8)
25-27	425,346 (14.8)	156,446 (16.3)	43,516 (16.4)	181,698 (16.5)	143,758 (21.0)	950,764 (16.1)
21-24	201,916 (7.0)	77,074 (8.0)	22,160 (8.3)	83,141 (7.5)	93,783 (13.7)	478,074 (8.1)
15-20	105,898 (3.7)	40,685 (4.2)	12,151 (4.6)	39,311 (3.6)	58,804 (8.6)	256,849 (4.4)
1-14	89,023 (3.1)	28,100 (2.9)	7,660 (2.9)	24,280 (2.2)	37,799 (5.5)	186,862 (3.2)
Unknown	459,356 (16.0)	115,367 (12.0)	62,736 (23.6)	138,419 (12.6)	71,346 (10.4)	847,224 (14.4)

SD, standard deviation.
[a]Mild inflammation includes gingivitis, stomatitis, mucositis, pericoronitis, and other unspecific inflammatory conditions.
[b]Periodontitis includes mild, advanced periodontitis, and periimplantitis.

Supplementary Table 6. Characteristics of all participants at first dental visits by number of teeth, in the cohort identified from the Swedish Dental Health Register, 2009-2016

**Characteristics**	**Number of teeth (number of individuals, %)**						
	28+	24-27	21-23	15-20	1-14	Unknown	Total
Total	3,169,668 (53.8)	950,764 (16.1)	478,074 (8.1)	256,849 (4.4)	186,862 (3.2)	847,224 (14.4)	5,889,441 (100)
Age at baseline							
Total	40.9 ± 15.1	55.2 ± 15.9	64.0 ± 15.3	72.1 ± 11.0	73.5 ± 12.3	39.6 ± 19.2	47.3 ± 18.9
>19 and <50	2,274,139 (71.7)	305,862 (32.2)	70,767 (14.8)	8,343 (3.2)	8,120 (4.3)	610,016 (72.0)	3,277,247 (55.6)
≥50 and <70	796,321 (25.1)	496,208 (52.2)	230,119 (48.1)	95,266 (37.1)	55,622 (29.8)	159,376 (18.8)	1,832,912 (31.1)
≥70	99,208 (3.1)	148,694 (15.6)	177,188 (37.1)	153,240 (59.7)	123,120 (65.9)	77,832 (9.2)	779,282 (13.2)
Calendar year of baseline							
2009-2012	2,750,959 (86.8)	899,373 (94.6)	460,405 (96.3)	249,219 (97.0)	178,109 (95.3)	695,706 (82.1)	5,233,771 (88.9)
2013-2016	418,709 (13.2)	51,391 (5.4)	17,669 (3.7)	7,630 (3.0)	8,753 (4.7)	151,518 (17.9)	655,670 (11.1)
Sex							
Male	1,610,435 (50.8)	427,782 (45.0)	207,114 (43.3)	112,313 (43.7)	85,533 (45.8)	426,938 (50.4)	2,870,115 (48.7)
Female	1,559,233 (49.2)	522,982 (55.0)	270,960 (56.7)	144,536 (56.3)	101,329 (54.2)	420,286 (49.6)	3,019,326 (51.3)
Education level							
Low	340,824 (10.8)	189,652 (19.9)	156,442 (32.7)	119,233 (46.4)	98,003 (52.4)	157,046 (18.5)	1,061,200 (18.0)
Medium	1,605,794 (50.7)	451,866 (47.5)	207,677 (43.4)	96,992 (37.8)	64,745 (34.6)	457,466 (54.0)	2,884,540 (49.0)
High	1,179,740 (37.2)	276,804 (29.1)	94,063 (19.7)	30,811 (12.0)	17,970 (9.6)	212,635 (25.1)	1,812,023 (30.8)
Unknown	43,310 (1.4)	32,442 (3.4)	19,892 (4.2)	9,813 (3.8)	6,144 (3.3)	20,077 (2.4)	131,678 (2.2)
Family income							
Low	926,869 (29.2)	262,031 (27.6)	148,266 (31.0)	93,098 (36.2)	77,404 (41.4)	321,209 (37.9)	1,828,877 (31.1)
Medium	1,013,514 (32.0)	327,165 (34.4)	173,764 (36.3)	97,233 (37.9)	69,000 (36.9)	259,979 (30.7)	1,940,655 (33.0)
High	1,229,285 (38.8)	361,568 (38.0)	156,044 (32.6)	66,518 (25.9)	40,458 (21.7)	266,036 (31.4)	2,119,909 (36.0)
Smoking-related diseases							
No	3,148,587 (99.3)	937,531 (98.6)	465,460 (97.4)	245,051 (95.4)	174,738 (93.5)	832,749 (98.3)	5,804,116 (98.6)
Yes	21,081 (0.7)	13,233 (1.4)	12,614 (2.6)	11,798 (4.6)	12,124 (6.5)	14,475 (1.7)	85,325 (1.4)
Drinking-related disorders and diseases							
No	3,101,694 (97.9)	929,680 (97.8)	465,661 (97.4)	248,558 (96.8)	178,774 (95.7)	814,706 (96.2)	5,739,073 (97.4)
Yes	67,974 (2.1)	21,084 (2.2)	12,413 (2.6)	8,291 (3.2)	8,088 (4.3)	32,518 (3.8)	150,368 (2.6)
Family history of pancreatic cancer							
No	3,137,574 (99.0)	933,002 (98.1)	460,496 (96.3)	241,581 (94.1)	173,783 (93.0)	833,058 (98.3)	5,779,494 (98.1)
Yes	11,140 (0.4)	5,095 (0.5)	2,589 (0.5)	1,341 (0.5)	917 (0.5)	2,415 (0.3)	23,497 (0.4)
Unknown	20,954 (0.7)	12,667 (1.3)	14,989 (3.1)	13,927 (5.4)	12,162 (6.5)	11,751 (1.4)	86,450 (1.5)
Dental health status							
Healthy	1,592,690 (50.2)	425,346 (44.7)	201,916 (42.2)	105,898 (41.2)	89,023 (47.6)	459,356 (54.2)	2,874,229 (48.8)
Caries	544,755 (17.2)	156,446 (16.5)	77,074 (16.1)	40,685 (15.8)	28,100 (15.0)	11,5367 (13.6)	962,427 (16.3)
Root canal infection	117,898 (3.7)	43,516 (4.6)	22,160 (4.6)	12,151 (4.7)	7,660 (4.1)	62,736 (7.4)	266,121 (4.5)
Mild inflammation[a]	634,980 (20.0)	181,698 (19.1)	83,141 (17.4)	39,311 (15.3)	24,280 (13.0)	138,419 (16.3)	1,101,829 (18.7)
Periodontitis[b]	279,345 (8.8)	143,758 (15.1)	93,783 (19.6)	58,804 (22.9)	37,799 (20.2)	71,346 (8.4)	684,835 (11.6)

SD, standard deviation.
[a]Mild inflammation includes gingivitis, stomatitis, mucositis, pericoronitis, and other unspecific inflammatory conditions.
[b]Periodontitis includes mild, advanced periodontitis, and periimplantitis.

Supplementary Table 7. Time-varying hazard ratios (HRs) with 95% confidence intervals (CIs) for pancreatic cancer according to dental health status and number of teeth, stratified by age at entry, in the cohort identified from the Swedish Dental Health Register, 2009-2016

**Parameters**	**>19 and <50 years**		**≥50 and <70 years**		**≥70 years**	
	**Cancer cases (%)**	**Crude model, HR (95% CI)**	**Cancer cases (%)**	**Crude model, HR (95% CI)**	**Cancer cases (%)**	**Crude model, HR (95% CI)**
Dental health status[a]						
Total	393 (100)		4,407 (100)		5,281 (100)	
Healthy	87 (22.1)	Ref.	640 (14.5)	Ref.	971 (18.4)	Ref.
Caries	50 (12.7)	0.86 (0.61-1.22)	442 (10.0)	0.98 (0.87-1.10)	663 (12.6)	1.02 (0.92-1.12)
Root canal infection	37 (9.4)	1.54 (1.05-2.27)*	249 (5.7)	1.09 (0.94-1.26)	267 (5.1)	1.03 (0.90-1.18)
Mild inflammation	111 (28.3)	0.99 (0.74-1.31)	951 (21.6)	0.97 (0.88-1.07)	1,151 (21.8)	1.03 (0.95-1.13)
Periodontitis	108 (27.5)	1.33 (1.00-1.77)*	2,125 (48.2)	1.24 (1.13-1.35)***	2,229 (42.2)	1.08 (1.01-1.17)*
Number of teeth[b]						
Total	380 (100)		4,178 (100)		4,578 (100)	
28+	260 (68.4)	Ref.	1,194 (28.6)	Ref.	396 (8.7)	Ref.
25-27	88 (23.2)	1.37 (1.07-1.74)*	1,317 (31.5)	1.14 (1.05-1.23)**	860 (18.8)	0.99 (0.88-1.11)
21-24	20 (5.3)	1.35 (0.86-2.13)	827 (19.8)	1.26 (1.15-1.38)***	1,197 (26.1)	1.06 (0.95-1.19)
15-20[c]	12 (3.1)	2.54 (1.42-4.54)**	495 (11.8)	1.61 (1.44-1.79)***	1,085 (23.7)	1.05 (0.93-1.18)
1-14	-	-	345 (8.3)	1.79 (1.58-2.02)***	1,040 (22.7)	1.22 (1.08-1.37)***

[a]Dental health status with the progressive diagnoses is treated as a time-varying exposure; due to frequent dental clinic visits for each participant, we kept the most severe diagnosis by calendar year after baseline, so that each has at most 8 records in the dataset (2009-2016).
[b]Categorical number of teeth is treated as a time-varying exposure, and fewer teeth with corresponding visit dates were ascertained after baseline.
[c]In the <50 age group, the subgroups having 15-20 and 1-14 teeth were combined as a subgroup of 1-20 teeth due to the small number of cancer cases.
*, *P* <0.05; **, *P* <0.01; ***, *P* <0.001.

References

1 Ljung, R., Lundgren, F., Appelquist, M., Cederlund, A. The Swedish dental health register - validation study of remaining and intact teeth. *BMC Oral Health* **19**, 116 (2019).

2 *Tillståndet och utvecklingen inom hälsooch sjukvård och tandvård*, https://www.socialstyrelsen.se/globalassets/sharepoint-dokument/artikelkatalog/ovrigt/2021-3-7307.pdf (Accessed Aug 19, 2021).

3 Haut, E. R., Pronovost, P. J. Surveillance Bias in Outcomes Reporting. *JAMA* **305**, 2462-2463 (2011).

4 Stolzenberg-Solomon, R. Z., Dodd, K. W., Blaser, M. J., Virtamo, J., Taylor, P. R., Albanes, D. Tooth loss, pancreatic cancer, and Helicobacter pylori. *Am J Clin Nutr* **78**, 176-181 (2003).

5 Michaud, D. S., Joshipura, K., Giovannucci, E., Fuchs, C. S. A prospective study of periodontal disease and pancreatic cancer in US male health professionals. *J Natl Cancer Inst* **99**, 171-175 (2007).

6 Michaud, D. S., Liu, Y., Meyer, M., Giovannucci, E., Joshipura, K. Periodontal disease, tooth loss, and cancer risk in male health professionals: a prospective cohort study. *Lancet Oncol* **9**, 550-558 (2008).

7 Hiraki, A., Matsuo, K., Suzuki, T., Kawase, T., Tajima, K. Teeth loss and risk of cancer at 14 common sites in Japanese. *Cancer Epidemiol Biomarkers Prev* **17**, 1222-1227 (2008).

8 Arora, M., Weuve, J., Fall, K., Pedersen, N. L., Mucci, L. A. An exploration of shared genetic risk factors between periodontal disease and cancers: a prospective co-twin study. *Am J Epidemiol* **171**, 253-259 (2010).

9 Michaud, D. S., Izard, J., Wilhelm-Benartzi, C. S., You, D. H., Grote, V. A., Tjonneland, A. *et al.* Plasma antibodies to oral bacteria and risk of pancreatic cancer in a large European prospective cohort study. *Gut* **62**, 1764-1770 (2013).

10 Wen, B. W., Tsai, C. S., Lin, C. L., Chang, Y. J., Lee, C. F., Hsu, C. H. *et al.* Cancer risk among gingivitis and periodontitis patients: a nationwide cohort study. *Q J Med* **107**, 283-290 (2014).

11 Hwang, I. M., Sun, L. M., Lin, C. L., Lee, C. F. Kao, C. H. Periodontal disease with treatment reduces subsequent cancer risks. *Q J Med.* **107**, 805-812 (2014).

12 Chang, J. S., Tsai, C. R., Chen, L. T., Shan, Y. S. Investigating the Association Between Periodontal Disease and Risk of Pancreatic Cancer. *Pancreas* **45**, 134-141 (2016).

13 Huang, J., Roosaar, A., Axell, T., Ye, W. A prospective cohort study on poor oral hygiene and pancreatic cancer risk. *Int J Cancer* **138**, 340-347 (2016).

14 Michaud, D. S., Kelsey, K. T., Papathanasiou, E., Genco, C. A., Giovannucci, E. Periodontal disease and risk of all cancers among male never smokers: an updated analysis of the Health Professionals Follow-up Study. *Ann Oncol* **27**, 941-947 (2016).

15 Nwizu, N. N., Marshall, J. R., Moysich, K., Genco, R. J., Hovey, K. M., Mai, X. *et al.* Periodontal Disease and Incident Cancer Risk among Postmenopausal Women: Results from the Women's Health Initiative Observational Cohort. *Cancer Epidemiol Biomarkers Prev* **26**, 1255-1265 (2017).

16 Fan, X., Alekseyenko, A. V., Wu, J., Peters, B. A., Jacobs, E. J., Gapstur, S. M. *et al.* Human oral microbiome and prospective risk for pancreatic cancer: a population-based nested case-control study. *Gut* **67**, 120-127 (2018).

17 Michaud, D. S., Lu, J., Peacock-Villada, A. Y., Barber, J. R., Joshu, C. E., Prizment, A. E. *et al.* Periodontal Disease Assessed Using Clinical Dental Measurements and Cancer Risk in the ARIC Study. *J Natl Cancer Inst* **110**, 843-854 (2018).

18 Jordão, H. W., McKenna, G., McMenamin Ú, C., Kunzmann, A. T., Murray, L. J., Coleman, H. G. The association between self-reported poor oral health and gastrointestinal cancer risk in the UK Biobank: A large prospective cohort study. *United European Gastroenterol J.* **7**, 1241-1249 (2019).
19 Lee, K., Lee, J. S., Kim, J., Lee, H., Chang, Y., Woo, H. G. *et al.* Oral health and gastrointestinal cancer: A nationwide cohort study. *J Clin Periodontol* **47**, 796-808 (2020).
20 Zhang, X., Liu, B., Lynn, H. S., Chen, K., Dai, H. Poor oral health and risks of total and site-specific cancers in China: A prospective cohort study of 0.5 million adults. *eClinicalMedicine* **45** (2022).
21 Hujoel, P. P., Drangsholt, M., Spiekerman, C., Weiss, N. S. An exploration of the periodontitis-cancer association. *Ann Epidemiol* **13**, 312-316 (2003).
22 Ahn, J., Segers, S., Hayes, R. B. Periodontal disease, Porphyromonas gingivalis serum antibody levels and orodigestive cancer mortality. *Carcinogenesis* **33**, 1055-1058 (2012).
23 Ansai, T., Takata, Y., Yoshida, A., Soh, I., Awano, S., Hamasaki, T. *et al.* Association between tooth loss and orodigestive cancer mortality in an 80-year-old community-dwelling Japanese population: a 12-year prospective study. *BMC Public Health* **13**, 814 (2013).
24 Heikkilä, P., But, A., Sorsa, T., Haukka, J. Periodontitis and cancer mortality: Register-based cohort study of 68,273 adults in 10-year follow-up. *Int J Cancer* **142**, 2244-2253 (2018).
25 Chung, P. C., Chan, T. C. Association between periodontitis and all-cause and cancer mortality: retrospective elderly community cohort study. *BMC Oral Health* **20**, 168 (2020).
26 The Dental and Pharmaceutical Benefits Agency. *Föreskrifter om ändring i Tandvårds- och läkemedelsförmånsverkets föreskrifter och allmänna råd (TLVFS 2008:1) om statligt tandvårdsstöd,* https://www.tlv.se/download/18.1c9ccc53176e89932171fccd/1610445393307/hslf_fs 2020_28.pdf (Accessed Apr 16, 2022).
27 Bergman, D., Hagström, H., Capusan, A. J., Mårild, K., Nyberg, F., Sundquist, K. *et al.* Incidence of ICD-Based Diagnoses of Alcohol-Related Disorders and Diseases from Swedish Nationwide Registers and Suggestions for Coding. *Clin Epidemiol* **12**, 1433-1442 (2020).
28 Ye, W., Lagergren, J., Weiderpass, E., Nyren, O., Adami, H. O., Ekbom, A. Alcohol abuse and the risk of pancreatic cancer. *Gut* **51**, 236-239 (2002).
